# Letter to the editor regarding “let’s doff: a gown conservation strategy for multidrug-resistant organism colonization during the COVID-19 pandemic and beyond” by Rowe et al

**DOI:** 10.1017/ash.2025.10138

**Published:** 2025-09-17

**Authors:** Mahdee Saleh

**Affiliations:** Lorn and Islands Hospital, Oban, UK

The recent study by Rowe et al.^1^ provides timely, real-world insight into the enduring debate around contact precautions (CP) for asymptomatic carriers of methicillin-resistant *Staphylococcus aureus* (MRSA) and vancomycin-resistant enterococci (VRE). By analyzing healthcare-associated infection (HAI) outcomes across three time periods, before, during, and after the COVID-19 pandemic, the authors offer an important conclusion: modifying CP to conserve gowns during the pandemic did not result in increased HA-MRSA, HA-VRE, or *Clostridioides difficile* (HA-CDI) infections.

We commend the authors for producing a robust multicenter data set and applaud the clarity with which they contextualize their results. However, we believe this study also presents an opportunity to go further, beyond retrospective validation, and actively reassess the value, role, and sustainability of CP in routine clinical care.

Contact precautions, as currently defined by the CDC’s 2007 guideline, include gloves and gowns for all room entries when caring for patients colonized or infected with MDROs.^2^ The evidence base questioning CP for asymptomatic MDRO colonization continues to grow. As noted by Rowe et al., a 2018 meta-analysis^3^ and subsequent reviews^4^ found little infection control benefit from CP in these contexts. Simultaneously, harms to patients, from reduced healthcare worker contact to heightened anxiety and medical errors, are well documented.^5^

Yet even as evidence mounts, many institutions revert to CP “by default.” This inertia deserves re-examination, particularly given the significant economic and environmental costs. PPE accounted for £15 billion in UK NHS expenditure during the pandemic,^6^ and gowns are the single highest contributors to PPE carbon emissions in healthcare.^7^ These externalities are rarely included in infection control cost-benefit analyses, yet they must be, especially if they reframe CP as a high cost, low value practice.

Rowe et al also correctly identify the absence of PPE compliance audits as a limitation. However, this also highlights a systemic issue: CP adherence is assumed, not measured. Numerous studies show PPE protocol fatigue, incorrect doffing, and inconsistent glove use as common challenges even before COVID-19.^8^ A logical next step would be integrating real-time audit mechanisms alongside infection surveillance to better correlate policy, practice, and outcome.

The pandemic offered an unplanned but invaluable natural experiment: mass CP de-implementation in real time. Like others (eg, Johns Hopkins and University of California systems) who observed no significant increase in HAIs after reducing CP use,^9,10^ Rowe et al.’s findings support a broader reimagining of isolation protocols.

To capitalize on this, professional societies such as SHEA and HICPAC could offer updated, tiered CP guidance:

- For symptomatic infection: maintain CP.
- For asymptomatic colonization: evaluate local prevalence, resource strain, and patient risk factors.
- For low-risk units or end-of-life care: consider standard precautions only.

This study should not be viewed simply as justification for a pandemic-era workaround—it’s a springboard for permanent, thoughtful reform. BJC HealthCare’s decision to modify MDRO isolation policies long-term is commendable. Others should follow, not just to improve patient experience, but to align infection control with the pillars of antimicrobial stewardship, environmental sustainability, and equity.

In conclusion, Rowe et al.’s study contributes more than a retrospective validation of modified CP - it offers an invitation to challenge long-held assumptions. The postCOVID era demands we ask: not only do contact precautions work, but are they always worth it?
